# The Potential Role of Endothelial Cells in the Pathophysiology, Diagnosis, and Management of Acute Kawasaki Disease

**DOI:** 10.1016/j.cjco.2025.11.001

**Published:** 2025-11-12

**Authors:** Antonino Calvaruso, Laura Andreozzi, Lucia Paolini, Fiorentina Guida, Arianna Catelli, Eleonora Bellini, Eleonora Battelli, Marcello Lanari, Marianna Fabi

**Affiliations:** a Specialty School of Paediatrics, Alma Mater Studiorum, University of Bologna, Bologna, Italy; b Pediatric Emergency Unit, IRCCS Azienda Ospedaliero-Universitaria di Bologna, Bologna, Italy; c Department of Medical and Surgical Sciences, Alma Mater Studiorum, University of Bologna, Bologna, Italy

**Keywords:** Kawasaki disease, Endothelial cells, Coronary arteries, Endothelium, Endothelial damage, Endothelial injury

## Abstract

The vital role of the endothelium in Kawasaki disease (KD) has been clear for over 20 years. Endothelial cells (ECs) play a critical role in the pathophysiology of the disease, being the target of several molecular pathways resulting in EC damage, dysfunction, and potential detachment or transition to another cell phenotype. The alteration of endothelium health is crucial for the development of coronary artery damage. In children with KD, molecular components or cells are released from injured endothelium and can be measured in peripheral blood samples, suggesting several potential diagnostic and therapeutic implications. Novel diagnostic and therapeutic strategies focusing on ECs and on the pathways that result in endothelial damage have been investigated, but to date, the evidence is not yet strong enough to change clinical practice. Nonetheless, results are promising and provide a reliable basis to guide the design of translational research, as seen with statins, which have confirmed their beneficial effects on vascular integrity in later stages of KD, including in clinical studies. The purpose of this narrative review is to provide a comprehensive overview of the current knowledge on the role of ECs in the pathophysiology, diagnosis, and management of KD, and to synthesize the available evidence to guide further research aimed at confirming the viability of these new strategies.

## Résumé

Le rôle essentiel de l'endothélium dans la maladie de Kawasaki (MK) est connu depuis plus de 20 ans. Les cellules endothéliales (CE) jouent un rôle primordial dans la physiopathologie de la maladie, car elles sont la cible de plusieurs voies moléculaires entraînant des lésions des CE, une dysfonction et un possible détachement ou une transition vers un autre phénotype cellulaire. L'altération de l'intégrité endothéliale est cruciale dans la survenue de lésions des artères coronaires. Chez les enfants atteints de la MK, des composants moléculaires ou des cellules sont libérés par l'endothélium endommagé et peuvent être mesurés dans des échantillons de sang périphérique, ce qui suggère plusieurs implications diagnostiques et thérapeutiques potentielles. De nouvelles stratégies diagnostiques et thérapeutiques axées sur les CE et sur les voies menant aux lésions endothéliales ont été étudiées, mais à ce jour, les preuves ne sont pas encore suffisamment solides pour modifier la pratique clinique. Néanmoins, les résultats sont prometteurs et fournissent une base fiable pour orienter la conception de la recherche translationnelle, comme on l'a vu avec les statines, qui ont confirmé leurs effets bénéfiques sur l'intégrité vasculaire aux stades avancés de la MK, y compris dans des études cliniques. L'objectif de cette revue narrative est de fournir un aperçu complet des connaissances actuelles sur le rôle des CE dans la physiopathologie, le diagnostic et la prise en charge de la MK, et de synthétiser les données disponibles afin d'orienter les recherches futures visant à confirmer la viabilité de ces nouvelles stratégies.

Kawasaki disease (KD) is an acute, self-limiting, febrile illness affecting primarily children aged < 5 years. The incidence of KD in northeast Asian countries, such as Japan, South Korea, China, and Taiwan, is 10-30 times higher than it is in Europe and the US^1^^,^^2^—equal to 18-25 cases per 100,000 vs 10-15 cases per 100,000 children aged < 5 years, respectively.^3^

**Figure 1:**
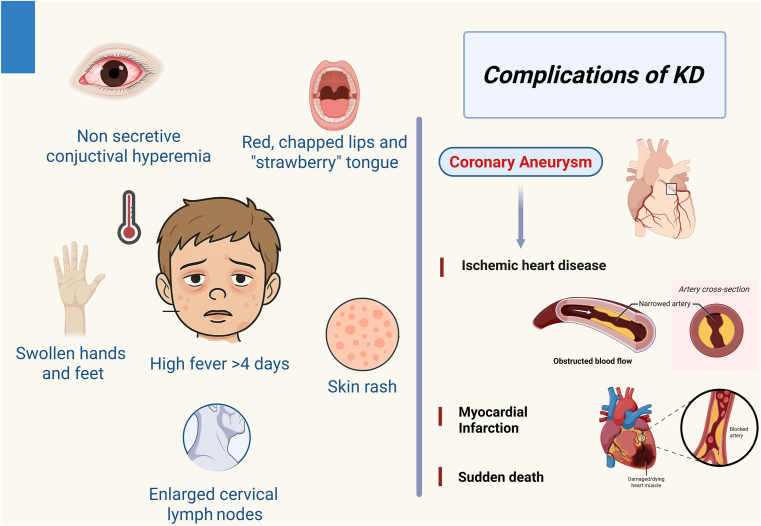
Diagnostic clinical criteria for Kawasaki disease and its main complications.

KD is the leading cause of acquired heart disease in children in high-income countries, due to its major complication—coronary involvement. Coronary injury can lead to permanent coronary artery damage, such as coronary aneurysms, and to long-term sequelae, such as myocardial infarction, ischemic heart disease, and sudden death. Coronary artery lesions (CALs) during the acute stage can be classified as dilatation and aneurysms, based on their dimensions indexed for body size. CALs can be transient when they resolve by 6-8 weeks after fever onset, or persistent, and thus can potentially evolve over time after their onset. When coronary injury occurs, endothelial damage predisposes patients to thrombus formation, eventually occluding the vessel.^4^ Alternatively, the arterial wall can remodel into further dilation or stenosis due to fibroblastic proliferation.^4^ The mainstay treatment is intravenous immunoglobulin (IVIG) infusion administered in the acute phase of KD to halt inflammation. However, the diagnosis of KD still relies on the combination of clinical manifestations, such as mucositis, conjunctival hyperemia, skin rash, alteration of extremities, and lymphadenopathy, in addition to fever persisting for more than 4 days^1^ (Fig. 1).

However, clinical diagnostic criteria commonly overlap with those of pediatric infectious diseases, and, especially when symptoms are minimal, KD diagnosis and treatment can be delayed, increasing the risk of CALs. Although the precise mechanism of action of high-dose IVIG in KD remains incompletely defined, emerging evidence points to both innate- and adaptive-immune modulation. In particular, IVIG administration is followed by a marked reduction of circulating neutrophils— including interleukin (IL)-1β-expressing mature neutrophils—together with an increased lymphocyte proportion and suppressed IL-1β release in KD patients.^5^ Moreover, Franco et al. showed that IVIG triggers the expansion of immunoglobulin (Ig)G-Fc-specific regulatory T cells (Tregs) in KD patients who have not developed coronary artery lesions, suggesting a role for Fc-dependent induction of immune regulation.^6^ The generalized modulatory and suppressive effects of IVIG on immune function lead to cessation of fever,^7^ improvement in the patient's clinical condition, and a reduced risk of coronary involvement. Without proper therapy, about 25% of KD patients develop CALs, but the risk decreases to 4% when IVIG is administered before day 10 of fever.^2^ About 10% of patients can be IVIG-resistant, meaning that fever persists or reappears at least 36 hours after the completion of IVIG infusion. IVIG unresponsiveness increases the risk of coronary injury.^8^

Endothelial cells (ECs) constitute the inner linings of blood vessels and are therefore directly exposed to the components and cells of the blood. They have a wide spectrum of functions: ECs contribute to the control of vascular tone and regional blood flow, inflammatory cell and blood fluidity, platelet adhesion and aggregation, and leukocyte function; they also play a role in the balance of coagulation and regulation of immune response, inflammation, and angiogenesis.^9^ The endothelium serves, therefore, as the interface between the circulating inflammatory cells and vascular media or adventitia, which is the first target of inflammatory attacks during the early stage of KD.

Many gaps remain in the understanding of KD pathogenesis, pathophysiology, diagnosis, and treatment. Given that KD outcomes can be severe, the need to fill these knowledge gaps, to improve diagnostic tools and therapeutic management, is urgent. In the era of the use of accessible body fluids to identify diseases, liquid biopsy, which samples body fluids, particularly molecular components or cells released from body tissues, can represent an alternative to tissue biopsies for studying diseases. Given that ECs are among the first cells to come into contact with circulating inflammatory mediators, the products of endothelial damage are expected to be released in the bloodstream.^10^ In this regard, ECs can represent a promising target for the investigation of vascular injury in KD and potentially for improving its management.^11^ In this paper, we discuss the current understanding of the potential role of ECs, focusing on the pathophysiology, the diagnostic process, and the therapeutic approaches used with KD. Table 1 summarizes the role of ECs in KD pathophysiology, diagnosis, and management.

**Table 1:** Summarizing the role of endothelial cells (ECs) in the pathophysiology, diagnosis, and management of Kawasaki disease (KD)

	Mechanism description
**KD pathophysiology**	
**Inflammation**	Intense immune-inflammatory response causes damage to ECs, activating cytokines, adhesion molecules, and growth factors, leading to dysfunction and lesions.^13^^,^^14^
**Pyroptosis and inflammasome**	Programmed cell death (pyroptosis) and NLRP3 inflammasome activation are key pathologic features in KD immune-induced inflammation. In KD mouse models, inhibiting NLRP3 reduces inflammation and improves vascular function.^17^, ^18^, ^19^, ^20^
**Autophagy**	Evidence is controversial. Several studies found a role of autophagy in the stimulation of inflammatory responses, but when the inflammatory reaction is overactive and uncontrolled, autophagy can be detrimental.^22^
**EndoMT and EMT**	Inflammation promotes EndoMT and EMT, transforming ECs into myofibroblasts that compromise vascular integrity. Decreased KLF4 and miR-483 lead to increased CTGF, favouring the loss of endothelial integrity.^26^^,^^29^
**NO system**	Most CECs in KD patients express iNOS but not eNOS, which is displayed in healthy controls. iNOS is found in ECs of coronary arteries with aneurysms, and its expression increases with the rise in CECs, particularly in the acute and subacute phases, suggesting a possible role in vascular damage and persistent inflammation.^36^, ^37^, ^38^, ^39^
**RAGE**	The RAGE protein, mediated by S100A12, contributes to neutrophil and monocyte infiltration in the vascular wall; elevated levels are observed in subacute and chronic KD stages and in IVIG-resistant patients, indicating prolonged inflammation.^40^, ^41^, ^42^
**EMPs**	EMPs, released from activated or apoptotic endothelial cells, are released into the bloodstream in response to proinflammatory molecules (TNF-alpha, cytokines, and reactive oxygen species) and seem to be correlated with endothelial dysfunction measured by flow-mediated dilation.^45^^,^^47^, ^48^, ^49^
**KD diagnosis**	
**CECs**	CECs are mature endothelial cells detached from the intima; CEC levels increase in diseases with endothelial dysfunction, including KD, with higher levels in those presenting CALs.^30^^,^^36^^,^^59^
**EPCs**	EPCs are nonhematopoietic cells that promote endothelial repair and reduce dysfunction; they activate in response to vascular injury. Levels increase in KD, though their functionality may be reduced.^16^^,^^59^
**EMPs**	EMPs are found in high amounts in KD, especially in patients with CALs; they may serve as potential diagnostic biomarkers for KD.^75^^,^^76^
**Soluble markers**	NO and adhesion proteins (eg, E-selectin) are promising for KD diagnosis and monitoring; NO levels are elevated in patients with coronary involvement.^36^^,^^78^, ^79^, ^80^, ^81^)
**KD therapy**	
**Standard treatment**	Although IVIGs seem to have a role in inhibiting ECs and proliferation, their beneficial effect might be incomplete, due to the finding that CECs increase, despite IVIG administration, from the acute to the subacute stage of the disease. ASA modulates the production of proinflammatory and pro-resolving mediators in ECs, modulating inflammation at the endothelial-leukocyte interface.^84^, ^85^, ^86^, ^87^
**Anakinra and TNF-alpha antagonist**	Anakinra, an IL-1Ra, is one of the suggested treatments for IVIG-resistant patients, due to its efficacy in reducing the incidence of CALs. Preclinical studies showed that the loss of IL-1 signalling attenuates EndoMT, potentially reducing coronary lesion formation. Infliximab, a TNF-alpha antagonist, is another biologic drug proven to have beneficial effects for IVIG-resistant patients. Its efficacy could be due to high TNF-alpha levels in KD sera. TNF-alpha seems to activate ECs to express some molecules, leading to increased endothelial-leukocyte cell interactions, and to stimulate inflammatory pathways, mitochondrial alteration, and ROS accumulation.^89^^,^^91^^,^^95^, ^96^, ^97^
**NFAT/FOXO4 pathway**	The NFAT/FOXO4 pathway regulates endothelial health; inhibiting NFAT reduces inflammation and may be a therapeutic strategy for KD.^98^
**Sirt1-NFkB pathway**	Forsythoside B reduces cardiovascular inflammation in KD by activating SIRT1 and inhibiting NFκB; it has a protective role for myocardial tissue in KD models.^17^^,^^100^
**KLF4-miR-483 axis and statins**	Statins improve endothelial function, reduce oxidative stress, and inhibit thrombogenic response. One of their mechanisms of action is the activation of the KLF4-miR-483 axis, which decreases CTGF in KD ECs.^28^^,^^101^^,^^108^, ^109^, ^110^

ASA, acetylsalicylic acid; CAL, coronary artery lesions; CEC, circulating endothelial cell; CTGF, connective tissue growth factor; EC, endothelial cells; EMP, endothelial derived microparticles; EMT, epithelial-mesenchymal transition; EndoMT, endothelial-mesenchymal transition; eNOS, endothelial NO synthase; EPC, endothelial progenitor cell; FOXO4, Forkhead box protein O1; IL, interleukin; IL-1Ra, IL-1 receptor antagonist; iNOS, inducible NO synthase; IVIG intravenous immunoglobulin; KD, Kawasaki disease; KLF-4, Kruppel-like factor 4; miR, microRNA; NFAT, nuclear factor of activated T cells; NLRP3, NOD-, LRR-, and pyrin domain-containing protein 3; NO, nitric oxide; RAGE, receptor for advanced glycation end product; ROS, reactive oxygen species; SIRT1, sirtuin 1; SPM, pro-resolving mediators; TNF, tumour necrosis factor.

## Role of ECs in KD Pathophysiology

Despite the fact that KD was first described in the English-language literature almost 50 years ago,^12^ its pathogenesis and pathophysiology are not yet fully clarified.

Current evidence on KD pathogenesis suggests that activation of the immune system in genetically predisposed subjects, triggered by an unknown environmental factor, leads to intense systemic inflammation and vasculitis of small and medium-sized vessels.^2^

The systemic inflammation is due to the activation of both innate and adaptive immunity, leading to the release of cytokines by different cells, including monocytes and macrophages, ultimately causing endothelial injury^13^^,^^14^. Endothelial damage, activation, and remodelling are mirrored by increased *in situ* levels of pro-inflammatory cytokines, adhesions molecules, and growth factors.

*In vitro* and *in vivo* EC models may serve as the lens to focus on the molecular mechanisms triggered by inflammation that leads to endothelial damage and dysfunction, which in turn are mirrored by the level of ECs in the bloodstream. The finding of ECs in the peripheral bloodstream can be explained as resulting from 2 mechanisms—vascular injury and the attempt to repair the vessel. The enumeration of circulating endothelial cells (CECs) can contribute to investigating vascular injury, with the hypothesis that increased levels of CECs result from the detachment of ECs from the site of damage.^15^

The enumeration of endothelial progenitor cells (EPCs) can, on the other hand, mirror the attempt to repair the vessel. EPCs have a high proliferative potential in that they originate from the bone marrow and are mobilized by vascular-endothelial growth factor (VEGF) to reach the site of endothelial injury to repair the damage.^16^

**Figure 2:**
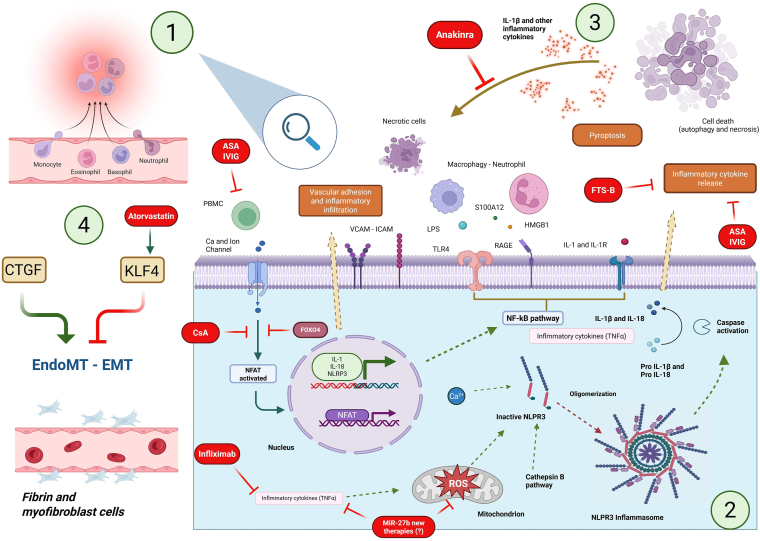
(**Green circle 1**) necrotic cells and peripheral blood mononuclear cells (PBMCs) trigger autophagy of human coronary artery endothelial cells (HCAECs) and release molecules such as high mobility group box 1 (HMGB1) and S100 calcium binding protein A12 (S100A12). These molecules interact with pattern recognition receptors, including the receptor for advanced glycation end-products (RAGE) and toll-like receptor 4 (TLR4), promoting downstream inflammatory signalling. (**Green circle 2**) This activation leads to the production of reactive oxygen species (ROS), lysosomal disruption, and cathepsin B release, triggering NOD-, LRR- and pyrin domain-containing protein 3 (NLRP3) inflammasome assembly. (**Green circle 3**) Subsequent caspase-1 activation facilitates the cleavage and secretion of pro-inflammatory cytokines, including interleukin-1β (IL-1β) and interleukin-18 (IL-18). (**Green circle 4**) These cytokines further act on endothelial cells, inducing phenotypic changes such as endothelial-to-mesenchymal transition (EndoMT), and upregulation of adhesion molecules (vascular cell adhesion molecule [VCAM]-1, intercellular cell adhesion molecule [ICAM]-1), connective tissue growth factor (CTGF), and downregulating the transcription factor Kruppel-like factor 4 (KLF4). Endothelial activation also is associated with the release of endothelial microparticles (EMPs), small vesicles shed from activated or apoptotic endothelial cells. EMPs act as both biomarkers and effectors of vascular injury: they can propagate inflammatory signalling, modulate immune cell activation, and amplify vascular remodelling by transferring bioactive molecules to target cells. These processes contribute to endothelial dysfunction and the development of vasculitis, a hallmark of Kawasaki disease. The **green arrows** indicate positive regulation of the underlying mechanism, and **red arrows** indicate inhibition of the pathway. The **dashed yellow arrows** indicate the final effect. The **red box** represents a possible therapeutic approach in Kawasaki disease. IL-1 inhibitor. anakinra(); ASA, acetylsalicylic acid; CA, calcium; CsA, cyclosporine; EndoMT, endothelial-mesenchymal transition; EMT, epithelial-mesenchymal transition; FOXO4, Forkhead box protein O4; FTS-B, forsythoside B; ; anti-TNFα, infliximab; IL-1R, interleukin-1 receptor; IVIG, intravenous immunoglobulin; LPS, lipopolysaccharide; NF-κB, nuclear factor kappa-light-chain-enhancer of activated B cells; NFAT, nuclear factor of activated T cells; ; pro IL-1β / pro IL-18, inactive precursors of IL-1β and IL-18.

Both CECS and EPCS are discussed in more detail in the Diagnosis section. Endothelial injury has been investigated *in vitro* and *in vivo* in the attempt to partially clarify the molecular mechanisms of damage. The main mechanisms that may be involved in endothelial damage are summarized in Figure 2.

### Pyroptosis and inflammasomes

Pyroptosis and the activation of inflammasomes seem to have a central role in the pathophysiology of KD. Pyroptosis is a form of cell death with characteristics of both apoptosis and necrosis. Several pathways of pyroptosis activation have been investigated in KD models. Among them, the sirtuin 1 (SIRT1)-nuclear factor (NF)κB signalling pathway has been studied. Through its regulatory function on the NFκB pathway, SIRT1 promotes the resolution of inflammation and limits pathologic myocardial remodelling, eventually limiting cardiovascular inflammatory disorders and providing a potential protective mechanism against myocardial damage in KD.^17^ Notably, NFkB has opposite functions, inducing proinflammatory responses.^18^

Inflammasomes are multiprotein signaling complexes that are essential for the inflammatory response to infections or damage. The best characterized of these is NOD-, LRR-, and pyrin domain-containing protein 3 (NLRP3) inflammasome. NLRP3 activation results in the release of proinflammatory cytokines, such as IL-1β and IL-18. Jia et al.^19^ demonstrated the pyroptosis of ECs: EC pyroptosis, and thus EC death, is activated by increased levels of high-mobility group box (HMGB) released by immune or necrotic cells, triggering, via cathepsin B, the activation of NLRP3 inflammasome. The latter, in turn, is activated in the *Lactobacillus Casei* cell-wall extract (LCWE)-induced KD mouse model in the endothelium of the coronary arteries. Notably, Xu et al. demonstrated that in an *in vivo* animal KD vasculitis model, the inhibition of the NLRP3-dependent pyroptosis pathway by human umbilical cord-derived mesenchymal stem cells (Huc-MSCs) reduces inflammation and improves vascular endothelial function, thereby increasing the capacity of the damaged ECs to proliferate and migrate, and inhibiting their apoptosis.^20^

### Autophagy

Autophagy is a biological mechanism enabling the degradation of intracellular components; it is involved in many processes, including inflammation and endothelial injury. Autophagy is a homeostatic mechanism designed to maintain normal cardiovascular function and morphology.

Growing evidence indicates that autophagy is involved in endothelial dysfunction and vascular endothelial injury. However, controversial findings were documented in cardiac diseases: autophagy seems to exert a beneficial effect on myocardial ischemia and reperfusion injury,^21^ but it also promotes cell death and atherosclerosis.^22^ Several studies found that autophagy plays a role in the stimulation of inflammatory responses, but when the inflammatory reaction is overactive and uncontrolled, autophagy can be detrimental.^22^ Human coronary artery ECs (HCAECs) are ECs in the heart, and they are used in adhesion studies—as a reflection of endothelial function—to investigate thrombogenicity and endothelial recovery in stent stenosis in patients with coronary artery disease (CAD). Although the pathophysiology of CAD and KD are different, HCAECs were used by some authors to focus on KD pathology from the stromal cell perspective. In acute KD, peripheral blood mononuclear cells (PBMCs) trigger autophagy of HCAECs and the secretion of chemokines and proinflammatory factors, such as tumour necrosis factor (TNF)-alpha, IL-6, and IL-8, as well as the expression of adhesion molecules, such as intercellular and vascular cell adhesion molecules (ICAMs and VCAMs).^23^

NLR family pyrin domain-containing 1 (NLRP1) has pattern recognition receptors (PRRs) and plays a role in regulating autophagy. Kimura et al. showed that in *Candida albicans,* water-soluble fraction (CAWS)-induced coronary arteritis, which is a mouse model of Kawasaki disease in NOD1-deficiency models, autophagy was downregulated, leading to a more severe disease phenotype.^24^ Some particular haplotypes of their genes appeared to be partly involved in the pathogenesis of KD.^25^

Autophagy also has a synergic action with the TNF-alpha/NfκB pathway. The NfκB pathway is a crucial regulator of inflammation, immune response, and cell survival, and it is regulated by TNF-alpha and IL-1. Sequestosome 1 (SQSTM1) is a protein with pleiotropic effects that triggers the NFκB pathway and mediates protein degradation, cell death, and cell survival.^26^ Autophagy and SQSTM1 influence the endothelial-mesenchymal transition (EndoMT),^27^ the process that transforms ECs into myofibroblasts,^28^ altering the structure of the coronary arteries in KD.^4^ Anti-apoptotic genes were found to be less expressed in KD when compared to another inflammatory condition, named multisystem inflammatory syndrome in children (MIS-C),^26^ that shares clinical and laboratory findings with KD, but has different cardiac involvement, with myocardial inflammation rather than coronary injury.

### Endothelial-mesenchymal transition (EndoMT) and epithelial-mesenchymal transition (EMT)

CALs are characterized by a loss of integrity of the media, leading to aneurysm formation: the release of inflammatory mediators, such as metalloproteinases and connective tissue growth factor (CTGF), alters collagen structure, replacing it with myofibroblasts. Myofibroblasts can originate from different cells, including ECs, by different mechanisms—EndoMT and EMT.

Dysregulation of vascular homeostasis and mesenchymal transition was shown by He et al.^29^ Kruppel-like factor 4 (KLF4), an important regulator of endothelial health, induces microRNA (miR)-483, which suppresses CTGF expression. This protective mechanism is reduced in ECs incubated with sera from KD patients, leading to unsuppressed CTGF. Both CTGF and KLF4 are crucial for vascular health, but they have opposite functions. CTGF contributes to EndoMT and EMT that occur in KD coronary artery walls, favouring the proliferation of myofibroblastic cells. On the contrary, KLF4 can reverse EndoMT, as a key regulator of endothelial homeostasis. Higher levels of transcripts associated with EndoMT were shown by Kim et al. in ECs incubated with KD sera, compared to ECs incubated with MIS-C sera before treatment.^26^ The ECs incubated with KD sera expressed a higher level of transcripts associated with inflammation, cell apoptosis, and EndoMT and reduced production of nitric oxide (NO), a critical molecule in maintaining endothelial cell homeostasis, regulating permeability and vascular tone. All these findings could explain the release of a higher number of CECs into the blood circulation in KD^30^ vs in MIS-C patients, and the progression of EndoMT, via promotion of loss of vascular integrity.

### Nitric oxide (NO) system

NO can be synthesized by 3 isoforms of NO synthase (NOS)—endothelial (eNOS), inducible (iNOS), and neuronal (nNOS). The different isoforms have different effects and in normal conditions, eNOS is constitutively expressed by ECs, whereas iNOS is not; nNOS is a constitutive form^31^ and could influence endothelial health.^32^

In particular, eNOS is involved in the production of NO, which has protective effects on the endothelium. eNOS reduces vasoconstriction, platelet aggregation, leukocyte adhesion, and smooth muscle cell proliferation. On the other hand, iNOS expression is known to increase after inflammation, producing large amounts of NO that can have a detrimental effect on the vascular wall, eventually causing its degeneration.

nNOS is expressed in the brain, peripheral nerves, and kidneys^33^ and plays an important role in influencing local endothelial health.^34^ To date, no studies have been conducted on nNOS and KD, but recent evidence suggests that local nNOS-derived NO in coronary arteries and arterioles, possibly from nitrergic neurons innervating coronary vessels, regulates basal coronary blood flow in humans.^35^ Given the rare neurologic involvement in KD, including symptoms such as irritability, aseptic meningitis, facial nerve palsy, seizures, and encephalopathy, further studies are needed to clarify the potential role of nNOS in endothelial function, particularly in affected patients.

Yu et al.^36^ studied iNOS in peripheral blood leukocytes and CECs in KD patients before and after treatment. The authors showed increased iNOS expression in neutrophils, during the acute stage of KD, especially in patients with CALs, and in monocytes only during the subacute stage of the disease. In addition, iNOS-positive ECs were shown in 3 specimens of coronary arteries from KD patients with aneurysms, rather than eNOS, whereas in normal controls, ECs from coronary artery specimens displayed eNOS, not iNOS. These findings seem to suggest that during acute KD in ECs within KD-associated lesions, iNOS expression is upregulated and eNOS is downregulated, potentially contributing to endothelial dysfunction and vascular inflammation. Nevertheless, aneurysm formation results from not only from EC injury but also the subsequent neutrophil infiltration of the media, chemokine-mediated CD8^+^ T-cell recruitment, and vascular smooth muscle cell destruction, which compromise the arterial wall structure. The concomitant rise in CECs and iNOS expression likely reflects EC detachment secondary to this inflammatory damage, contributing to the progression of CALs.^36^

In mice models of KD, peroxinitrite and nitrotyrosine increased concurrently with aneurysm development, whereas they were absent in negative controls.^37^ Peroxinitrite results from the reaction of NO with the superoxide radical, and causes endothelial activation^38^ and cellular injury, and nitrotyrosine from NO toxicity. Of note, both iNOS and nitrotyrosine were in perivascular macrophages and coronary arterioles of treated mice, suggesting that NO is involved in coronary injury.^37^However, to date, insufficient evidence is available to determine the exact role of NO in the pathogenesis of CALs in KD. Khajoee et al. studied the association of eNOS and iNOS gene polymorphisms with CALs in a Japanese population, without finding any significant association when coronary damage occurred.^39^

### Receptor for advanced glycation end product (RAGE)

An additional mechanism of vascular damage is mediated by RAGE via S100A12, a protein present mostly in granulocytes. S100A12 has a proinflammatory effect favouring the infiltration of the vascular wall by neutrophils and later by monocytes, activating ECs and leukocytes and recruiting monocytes into the site of inflammation.^40^ Specifically, the complex S100A12- RAGE in leukocytes and ECs activates NFκB, which in turn induces the expression of various proinflammatory genes.^41^

The expression of RAGE is increased on CEC surfaces and continues to increase in the subacute and chronic phases of KD and in IVIG-resistant patients, through S100A12 stimulation, which in turn increases the release of proinflammatory cytokines. Also, the number of RAGE-CECs was higher in patients with CALs after the acute stage, probably reflecting a longer-lasting inflammation.^42^ Notably, the number of CECs remains high years after KD onset, suggesting persistent vascular damage in all patients, with and without CALs,^43^ and supporting ongoing subclinical vascular inflammation.

### Endothelial-derived microparticles (EMPs)

EMPs are secreted in the form of secretory vesicles from cell membrane when ECs are activated or are undergoing apoptosis: the imbalance of transmembrane enzymes breaks down the cytoskeletal fibers, modifying the cell morphology and causing a bulge at the level of the plasma membrane.^44^^,^^45^

Flow cytometry can isolate EMPs using surface markers related to their endothelial origin and to the process that causes their release, because surface markers are different based on their origin.^46^ EMPs contain biological materials from their parental cells and carry endothelial proteins, such as vascular endothelial cadherin, platelet endothelial cell adhesion molecule-1, ICAM-1, endoglin, and others.^45^

Proinflammatory molecules, such as TNF-alpha, cytokines, and reactive oxygen species (ROS), facilitate the rupture of the bulges^45^ from the plasma membrane, taking part of the cytoplasm, and EMPs are released into the bloodstream. EMPs therefore include membrane proteins and cytoplasm components of ECs. The number of EMPs is high in the acute and subacute stages of KD, and decreases during the convalescent stage, but remaining at higher levels than in healthy controls.^47^ These results were corroborated by Ding at al. who found an earlier peak and slower decrease in KD patients, compared to healthy controls, but a similar pattern in febrile patients.^48^ Nakaoka et al.^49^ documented a peculiar pattern in relation to CALs: EMPs significantly increased in KD with CALs during the acute phase after IVIG, and decreased during convalescence, similar to the pattern in patients without CALs. In addition, 2 specific microRNAs encapsulated in EMPs might contribute to the vasculitis leading to CALs, through regulation of of inflammatory cytokines. The levels of EMPs were correlated significantly with endothelial dysfunction measured by flow-mediated dilation, suggesting a role of EMPs in vascular damage.^48^ Taken together, these findings could reflect involvement of EMPs in vascular injury, but further investigation is needed to define whether EMPs derive from EC activation or apoptosis. However, 2 specific microRNAs encapsulated in EMPs were found to have potential involvement in the pathogenesis of CALs in KD patients. Specifically, hsa-miR-145-5p and hsa-miR-320a, preferentially expressed in CALs, were demonstrated to affect monocyte function and upregulate inflammatory cytokine, potentially influencing cytokine regulation and contributing to vasculitis-forming CALs.^49^

## Role of ECs in KD diagnosis

The diagnosis of KD remains challenging, as it does not have pathognomonic criteria but rather relies on clinical features.^1^ When < 4 clinical signs are present, laboratory tests can support the diagnosis—anemia, thrombocytosis and/or thrombocytopenia, hypertransaminasemia, hyponatremia, and increased inflammatory markers—but these altered laboratory values are quite common in febrile children. In these cases, diagnosis may be delayed, and so may the treatment, potentially complicating outcomes. Therefore, the need is clear for innovative diagnostic methods to detect KD at an early stage.

As endothelial dysfunction may lead to functional and structural alterations of coronary and systemic arteries, its early identification by serum biomarkers might be crucial in the diagnosis and management of cardiovascular short and long-term complications.

Endothelial health can be assessed in different ways, through noninvasive methods (such as flow-mediated dilation or Endo-PAT) or invasive methods, which primarily consist of laboratory-based markers of endothelial dysfunction, including cellular and cellular-derived biomarkers and soluble biomarkers.^50^

### CECs

CECs are promising biomarkers of endothelial dysfunction in KD patients. CECs are mature ECs of approximately 15-50 micrometers in diameter, which detach from the *tunica intima* of the vessel as a result of an alteration of endothelial homeostasis and can be enumerated in the peripheral blood.^15^

The pioneers of the concept of CECs were Biuvier and Lhadovec, who first described these circulating non-hematopoietic cells of possible endothelial origin, using Gimsa staining. The first studies were based on the morphologic identification of CECs,^15^ but nowadays, the most commonly used methods are immunomagnetic bead isolation and fluorescence flow cytometry.^51^

Several markers, such as S-endo-1, also known as P1H12 and CD14S-endo-1, also known as P1H12 and CD146, CD131, CD62e, CD54, CD106, and CD141, specific to ECs have been identified. The absence of CD133 distinguishes CECs from EPCs.^52^

Several hypotheses have been proposed regarding the potential mechanisms for EC detachment from endothelium, explaining their increase in KD patients. The increase could be due to irreversible endothelial apoptosis or necrosis, but it also may be due to the disruption of the endothelial tethering to the matrix, caused by the activation of proteases and/or the effects of the cytokine cascade.^15^^,^^53^

A number of studies suggest that CEC levels may reflect individual endothelial health.^54^, ^55^, ^56^, ^57^, ^58^ In healthy adult subjects, CEC plasma levels are set at 5-14 cells/mL.^54^ Limits regarding CECs include the lack of reference data for healthy children; and heterogeneous data have been published as different methods of analysis have been used to evaluate CECs in KD so far.^30^^,^^59^ Although to date, no evidence is available on the normal range of CEC values in healthy children, several studies have investigated the role of CECs in this age group. Many studies focus on children and adolescents with obesity, hypertension, and other features of metabolic syndrome,^60^, ^61^, ^62^ severe infectious diseases, cancer, and other diseases, including KD.^30^^,^^63^, ^64^, ^65^ Increased levels of CECs were found in a wide range of diseases associated with endothelial dysfunction and inflammation in both adults and children, including hypertension, diabetes, preeclampsia, and chronic kidney failure.^33^, ^34^, ^35^

Nakatani et al.^59^ documented higher CEC levels in 20 children with KD, compared to levels in 10 healthy controls. In addition, CEC levels were higher in patients with CALs and varied with the disease stage, reaching a peak during the subacute phase. The authors suggested a potential explanation, based on the fact that CALs usually form during this phase, when the necrotizing arteritis finds its highest expression.^2^

In line with that possibility, we found higher CEC levels in KD patients during the acute stage compared to those in MIS-C patients, with a significant increase during the KD subacute stage.^30^ CEC levels in MIS-C patients are lower than those in KD, during both the acute and subacute stages, suggesting a different endothelial injury and response to IVIG therapy in KD and MIS-C. The different patterns may partially explain the different cardiovascular outcomes of the 2 diseases. In addition, clusters and/or syncytia were found in 50% of KD patients. As syncytium is a multinucleate mass of cytoplasm deriving from cell fusion, syncytia are thought to occur after tissue lesion and inflammation occur, as opposed to normal conditions.^30^ In this regard, an important translational consequence would be to intensify treatment from the early stage in those patients with CECs showing cluster and/or syncytia.

Yu et al. found similar results, documenting higher CEC levels in KD patients vs controls, with a peak at 2 weeks after the onset of the disease. In addition, the number of CECs was higher in patients with CALs than in those without CALs.^36^

These findings seem to suggest a promising role of CECs in distinguishing KD from MIS-C, especially when monitoring the evolution of the diseases stages; they also contribute to identifying those patients at higher risk for CALs, in whom they could help in modulating treatment. On the other hand, a cutoff diagnostic value for CECs has not been defined so far.

Notably, CEC levels remain high years after KD, suggesting that they may represent a marker of persistent vascular damage in all patients, with and without CALs. This finding also indicates an ongoing subclinical vascular inflammation and suggests a potential role of CECs in the coronary artery remodelling process.^43^ However, more data are needed before CEC enumeration can be considered a prognostic marker in KD patients, and further studies are needed to evaluate their role in identifying patients at increased risk for adverse cardiovascular outcomes.

### EPCs

Similar to CECs, EPCs are another group of nonhematopoietic cells in the blood. EPCs were first described in 1997 by Asahara et al. as circulating cells, expressing both hematopoietic and endothelial cell markers, that differentiated *in vitro* into ECs.^66^ EPCs may be enumerated by the same methods used for CECs but expressing different surface markers. Among them, CD34, CD31, von Willebrand Factor (vWF), CD146, and CD144 are the most common.^44^ Although CECs and EPCs share several surface markers, their origins and functions are different. EPCs originate from bone marrow and are mobilized by the VEGF to reach the sites of endothelial lesions. These cells have a high proliferative potential, facilitate endothelial repair, and reverse endothelial dysfunction. They are being studied currently for their promising role as candidate cell sources for revascularization, due to their endothelial regenerative capacity in patients with ischemic heart disease and ischemic stroke.^44^^,^^67^^,^^68^

In KD, EPC levels increase during the subacute phase and are higher in children with CALs; and their appearance in the bloodstream follows the peak of CECs.^59^ The different timing might reflect the role of these 2 cells, as follows: the increase of CECs mirrors endothelial injury, and the increase of EPCs mirrors the following angiogenesis thanks to their highly proliferative potential.

Compared to controls, Xu et al. showed that levels of EPCs in KD were higher^16^ and were characterized by lower migratory, proliferative, and adhesive activities. Notably, functional capacities improved after treatment with IVIG and aspirin, with consensual reduction of TNF-alpha and high-sensitivity C-reactive protein. These results seem to suggest an effect of the treatment on EPC functions, potentially accelerating the repair of vascular injury and improving the cardiovascular prognosis of KD patients.^69^

### Endothelial-derived microparticles

EMP levels in plasma increase 10-fold in several cardiovascular and metabolic diseases, such as CAD, diabetes mellitus, metabolic syndrome, stroke, pulmonary hypertension, renal failure, heart failure, and rheumatic diseases.^46^^,^^70^, ^71^, ^72^, ^73^, ^74^ In KD, EMPs were found to be increased by 1.6 and 2.4-fold when compared to levels in non-KD febrile and healthy children, and were positively correlated with TNF-alpha and negatively correlated with albumin.^75^ Notably, Nakaoka et al. found a correlation between EPM levels and the severity of coronary lesions,^49^ suggesting that EMPs are a biomarker of endothelial cell damage and that they can be used as a tool to identify those patients at higher risk for CALs who could benefit from a more aggressive therapy.

In a rabbit model of coronary artery vasculitis, Dou et al.^76^ showed that EMPs are shed from plasma membranes. In KD, the EMP levels are higher compared to those in healthy controls, and especially in the group with CALs, suggesting a possible diagnostic value of these particles in KD patients, to identify patients at higher risk.^48^^,^^75^^,^^77^ However, no specific cutoff values have been published to date.

### Soluble markers

Several studies seem to support the use of soluble markers as a diagnostic tool for KD. Among them, NO has been widely investigated. Increased levels of iNOS-derived NO are responsible for alteration of vascular homeostasis and arterial wall degeneration. NO levels in peripheral blood were found to be higher in children with coronary involvement and lower after IVIG treatment.^36^^,^^78^, ^79^, ^80^ EPCs were shown to correlate with NO levels,^43^ showing the deep relationship between cellular and soluble markers.

Yoshimura et al. showed higher levels of NO produced by neutrophils in KD patients, compared to healthy controls, and also to non-KD febrile patients.^80^ These results confirm the close relationship between KD pathophysiology and NO, and they draw attention to the need to investigate the possible role of NO as a KD biomarker.

Several adhesion molecules also have been studied as soluble biomarkers for KD patients, such as E-selectin (CD62E), P-selectin (CD62P), ICAM-1 (CD54), and VCAM-1 (CD106).^81^ These adhesion molecules are one of the mechanisms responsible for the anchorage to the extracellular matrix and the maintenance of the structural integrity of the endothelium. Several hypotheses have been proposed to explain the mechanism underlying the detachment of ECs from the endothelium, including the loss of survival signals derived from anchorage to the extracellular matrix, resulting in EC apoptosis and detachment.^82^ Several additional soluble markers have been proposed for use in KD patients, but further studies are needed to confirm their applicability.

## Role of ECs in KD Management

### Standard treatment

The main pillar of KD treatment is high-dose IVIG during the acute stage, and acetylsalicylic acid (ASA) for 6-8 weeks from KD diagnosis for all patients, and for longer to lifelong for those with severe coronary lesions.^1^ ASA modulates inflammation and prevents thrombosis. Despite the fact that the precise mechanism of IVIG has not been clarified completely, it includes some key effects—neutralization of pathogenic autoantibodies, suppression of TNF-alpha, and modulation or reduction of both B- and T-cell function.^7^^,^^83^

Additional treatment with IVIG to be given at diagnosis is recommended in those patients at high risk for IVIG resistance and for coronary injury, in order to increase immunomodulation. No specific drug has been proven to be more effective than others, and usually steroids, immunosuppressants, and biologics are used according to a centre’s experience. Other treatments could be valid tools to limit chronic vascular wall progressive damage that leads to thrombosis and stenosis over years in patients with large and giant coronary aneurysms during the first phases of the disease.

According to Xu et al., IVIG seems to inhibit proliferation of ECs in a time-dependent and fully reversible manner, partially explaining the effect of IVIG in reducing coronary involvement, from 25% to < 5%.^2^^,^^84^ In addition, a possible role of IVIG in activating natural killer cells and circulating CD16+ cells has been found.^85^ This beneficial effect may be due to the inability of IVIG to inhibit interaction between natural killer cells and ECs.^85^ However, the finding of increased numbers of CECs, despite IVIG administration, from the acute to the subacute stage of the disease, suggests that IVIG alone does not completely restore endothelial damage in KD patients after IVIG infusion.^30^^,^^59^ As mentioned, in MIS-C patients, CECs tend to decrease after IVIG and corticosteroid administration, suggesting that steroids may play a role in halting endothelial damage in these patients.^30^

ASA modulates the production of proinflammatory and pro-resolving mediators (SPMs) in ECs without the need of other cells, such as neutrophils and macrophages. SPMs are a group of bioactive lipids that actively mediate the resolution of inflammation, and lipoxins are a family of them. The inability to produce SPMs leads to persistence of inflammation. ASA also stimulates the production of an isomer of lipoxins, thereby exerting its pleiotropic effect.^86^ ASA also inhibits cicloxigenase-1 expressed in ECs, further modulating inflammation at the endothelial-leukocyte interface.^87^

### Adjunctive treatment: Anakinra and TNF-alpha antagonist

Among biologic drugs, anakinra, an IL-1 receptor antagonist (IL-1Ra), has shown promising results in the management of IVIG-resistant forms of KD, with reports suggesting potential benefits in controlling inflammation and limiting coronary complications. Although no randomized clinical trials, to date, have demonstrated a reduction of CALs in children treated with anakinra, it is currently considered a potential option for IVIG-resistant patients in the KD guidelines^(.^^1^^,^^2^^,^^88^ In murine models, where EndoMT has been identified as a pivotal link between inflammatory stress and endothelial dysfunction in aortic aneurysm disease,^89^ the loss of IL-1 signaling appears to attenuate EndoMT, potentially reducing aneurysmatic lesion formation, and the inhibition of IL-1β seems to enhance EC autophagy.^89^ In experimental models of esophageal adenocarcinoma, EndoMT-related changes in human esophageal microvascular endothelial cells were inhibited by IL-1β and TGF-β2 gene silencing in cultured adenocarcinoma cells; on the other hand, IL-1β and TGF-β2 induced EndoMT.^90^ Similar results were shown in human intestinal microvascular endothelial cells (HIMECs), in which morphologic and phenotypical changes were consistent with EndoMT after IL-1β, TGF-β1, and TNF-alpha exposure.^91^ Taken together, these experimental mechanisms may help explain the observed antiinflammatory and vasculoprotective effects of IL-1 blockade in KD in murine models^92^ and in IVIG-resistant KD patients,^93^ although clinical confirmation of this remains limited.

Infliximab, a TNF-alpha antagonist, is another biologic drug proven to represent an effective therapy for IVIG-resistant KD.^1^^,^^2^ The Kawasaki Disease Comparative Effectiveness Trial (KIDCARE) study showed that infliximab resulted in a shorter duration of fever, a decreased need for additional therapy, less-severe anemia, and shorter hospitalization when used instead of a second infusion of IVIG in IVIG-resistant patients.^94^ TNF-alpha levels are increased in KD sera, potentially activating the ECs to express ICAM-1, VCAM-1, iNOS, and IL-1β, through an increase in endothelial-leukocyte cell interactions.^95^ TNF-alpha antagonists thus would limit EC injury, blocking the TNF-alpha pathway.

On the other hand, in experimental models, TNF-alpha-related effects, such as activation of inflammatory pathways, mitochondrial alteration, and ROS accumulation, were shown to be limited by miR-27b. MicroRNAs have a central role in halting inflammation and oxidative stress-related EC dysfunction.^96^ Specifically, miR-27 is a family of microRNAs that are highly expressed in ECs, counteracting the TNF-alpha-related effects of restoring mitochondrial redox state, function, and membrane polarization by targeting Forkhead box protein O1 (FOXO1). MiR-27b could thus represent a target for future therapies for endothelial health.^97^

### Nuclear factor of activated T cells (NFAT)/FOXO4 pathway

The NFAT signalling pathway is another promising therapeutic target in KD. NFAT2 is the most potent member of the NFAT family. Huang et al.^98^ used TNF-alpha to stimulate HCAECs and mimic vasculitis *in vitro*. The authors showed that FOXO4 contributes to the maintenance of endothelial homeostasis, binding NFAT promoter region, and subsequently inhibiting the signalling pathway. The same pathway was shown in a CAWS-induced KD vasculitis mouse model. Specifically, NFAT2 was upregulated, and FOXO4 was downregulated, in CAWS-induced heart tissues. Blocking NFAT2 significantly reduced CAWS-induced vasculitis; and finally, FOXO4 modulates CAWS-induced vasculitis through NFAT2, suggesting a KD-promoting effect of NFAT2 and the opposite effect of FOXO4.^98^

When NFAT2 is inhibited in FOXO4-knockout mice, inflammation and inflammatory infiltration decrease,^98^ and so the FOXO4/NFAT2 pathway may represent a novel target among the treatment options for KD. Additionally, drugs that inhibit NFAT, such as cyclosporine (CsA), might contribute to preventing the progression of inflammation in the arterial wall by blocking the infiltration of cytotoxic CD8+ T cells. In experimental models, ECs exposed to sera from KD patients treated with CsA exhibited reduced proliferation, angiogenesis, NFATc1 levels, and inflammatory molecule production, compared to those not treated with CsA. Thus, CsA could exploit its cytoprotective effects through the enhancement of endothelial homeostasis via the Ca2+/NFAT pathway.^98^ However, whether cyclosporine has protective effects on coronary arteries when calcineurin inhibitors are used in refractory KD cases remains controversial.^99^

### Sirt1-NFkB pathway

The Sirt1-NFkB pathway has been proven to contribute to pyroptosis: Sirt1 promotes the resolution of inflammation,^17^ whereas NFkB has opposite functions, inducing proinflammatory responses.^18^ In KD models, SIRT1 expression is reduced and NFκB expression is increased, leading to KD-induced cardiac injury.^17^ Forsythoside B (FTS-B), extracted from the Forsythia suspensa plant, acts on SIRT1-NFkB signaling pathway inflammation, inhibiting inflammatory markers such as TNF-alpha, IL-6, and IκB, and thus modulating NFκB activity. Another effect in the same model was the improvement of left ventricular function.^17^

Sirtuins can be activated by resveratrol, a powerful antioxidant and a cardioprotector molecule. According to Huang et al., resveratrol inhibited TNF-alpha-induced ICAM-1, iNOS, and IL-1β mRNA expression in HCAECs, by inducing autophagy, and thus leading to the attenuation of vascular endothelial inflammation.^100^ These authors suggest a possible role of resveratrol as an adjuvant therapy for KD patients, to potentially reduce CAL formation.

### KLF4-miR-483 axis and statins

Statins are known mostly because they lower cholesterol levels, but they also have a pleiotropic effect; thus, they improve endothelial function, reduce oxidative stress and inflammation, and inhibit the thrombogenic response.^101^ Based on this, statins currently are recommended when severe coronary injury occurs.^1^ Among the statins, atorvastatin has been studied in KD. Myofibroblast-like phenotype cells, spindle-shaped cells, are involved in coronary aneurysm formation: they participate in the recruitment of proinflammatory cells and secrete a number of mediators, such as IL-17, matrix metalloproteinases, and CTGF, leading to arterial wall damage.^18^^,^^29^ These cells may originate from multiple sources, such as vascular ECs by EndoMT, perivascular progenitor cells by proliferation, vascular smooth muscle cells by losing the differentiation marker smoothelin, and circulating or adventitial fibroblasts by EMT^(^102, 103, 104 Genetic association studies in KD patients have demonstrated that polymorphisms in the TGF-β pathway influence disease susceptibility and coronary artery aneurysm formation.^105^ CTGF is regulated by the TGF-β pathway, which can induce both EMT and EndoMT.^106^^,^^107^ At the molecular level, EndoMT is characterized by the induction of mesenchymal markers, as well as decreased EC markers, such as vascular endothelial cadherin and eNOS. An interesting finding is that EndoMT can be reversed by overexpression of KLF4, a master regulator of EC homeostasis and phenotype.^108^^,^^109^

Given that atorvastatin activates the KLF4-miR-483 axis, decreasing CTGF in KD ECs, the same molecule could attenuate EndoMT, potentially representing a precious tool to hinder the loss of vascular wall integrity.^28^ Another statin, pravastatin, has been shown to have positive effects on endothelial function and to reduce low-grade chronic inflammation in KD patients with medium-to-large coronary aneurysms after 6 months of treatment.^110^ Thus, statins seem to provide a therapeutic strategy to preserve vascular wall integrity in KD patients after the acute phase, especially in those children who have had coronary injury.

## Translational and Clinical Implications

Although remarkable progress has been made in elucidating the molecular mechanisms underlying endothelial injury in KD, the translational bridge from bench to bedside remains largely incomplete. Most of the current evidence derives from *in vitro* and animal models, and only a few mechanistic insights have been translated into established or investigational clinical interventions.

After decades from their discovery, the main therapies with established clinical application remain IVIG and ASA, both of which modulate endothelial inflammation through pleiotropic antiinflammatory and immunoregulatory pathways. Statins, though primarily lipid-lowering agents, are currently recommended in selected KD patients with persistent or giant coronary aneurysms because of their demonstrated endothelial-protective effects via the KLF4–miR-483–CTGF axis. These agents, therefore, represent the most-mature translational targets for endothelial protection in KD.

In this scenario, new therapeutic agents such as anakinra and infliximab have shown promising results in IVIG-resistant KD, supported by mechanistic data linking IL-1 and TNF-α signalling to endothelial inflammation, autophagy, and EndoMT. Nevertheless, their current use remains off-label or limited to high-risk and refractory cases, as robust randomized controlled trials demonstrating a reduction in CALs are still lacking. Cyclosporine represents another emerging option under investigation for IVIG-resistant cases, but its vascular-protective efficacy continues to be debated.

Several molecular pathways, including SIRT1–NFκB signalling, autophagy, EndoMT modulation, RAGE–S100A12, and NO synthase dysregulation, remain at a preclinical or hypothesis-generating stage. Although agents such as resveratrol and forsythoside B show endothelial-protective potential in experimental models, and circulating endothelial biomarkers appear promising for diagnosis and monitoring, these findings still require clinical validation before they can be integrated into clinical decision-making.

## Conclusions

Many gaps in the understanding of the pathophysiology, diagnosis, and management of KD still need to be filled. To date, several studies have confirmed that many interplaying pathways are involved in systemic and vascular injury through inflammation, involving many soluble and cellular factors. ECs play a central role in this process. On one hand, they are a target of immune cells and cytokine cascade, resulting in cell damage, dysfunction, and potential detachment. On the other hand, ECs go through a process change in cell phenotype, toward mesenchymal cells, modifying the coronary vascular morphology and leading to coronary artery injury.

Thus, considering the vital role of the endothelium in KD, research on ECs can help shed light on this complex mechanism, potentially assisting in the diagnostic process, and in the identification of those patients who have greater endothelial damage. In this regard, ECs also could guide personalized treatment with the aim of reducing EC levels, potentially reflecting the ongoing endothelial injury predisposing coronary arteries to morphologic alterations leading to aneurysms.

Novel diagnostic and therapeutic strategies focusing on ECs and on the pathways that result in endothelial damage have been investigated in experimental models, but in most cases, the evidence is not yet strong enough to change clinical practice. Nonetheless, results are promising and provide a reliable basis to guide the design of translational research. Among potential therapies, statins have been investigated in clinical studies, with positive results. Their beneficial antioxidant and anti-inflammatory effects on ECs, which promote EC homeostasis and block EndoMT, have been confirmed *in vivo* in later stages of KD.^1^

Although preclinical studies are an essential step in the journey toward new discovery and development, clinical research is needed to confirm the viability of these new potential strategies. This narrative review creates a comprehensive synthesis of the current knowledge on ECs’ role in the pathophysiology, diagnosis, and management of KD, providing a solid basis to guide future research.
